# Effects of enzymatically modified isoquercitrin in supplementary protein powder on athlete body composition: a randomized, placebo-controlled, double-blind trial

**DOI:** 10.1186/s12970-019-0303-x

**Published:** 2019-09-10

**Authors:** Naomi Omi, Hideki Shiba, Eisaku Nishimura, Sakuka Tsukamoto, Hiroko Maruki-Uchida, Masaya Oda, Minoru Morita

**Affiliations:** 1 Faculty of Health and Sport Sciences, University of Tsukuba, Tsukuba, Japan; 2 Physical Education Graduate School, University of Tsukuba, Tsukuba, Japan; 3 Health Science Research Center, Morinaga & Co., Ltd, Tokyo, Japan

**Keywords:** Rutin, Quercetin, Antioxidant, Muscle mass, Weight management, Resistance training

## Abstract

**Background:**

Enzymatically modified isoquercitrin (EMIQ), a water-soluble quercetin, has been shown to intensify muscle hypertrophy in mice. We investigated the effect of EMIQ in supplementary protein powder on athlete body composition.

**Methods:**

Forty Japanese males who played American football (age: 19.8 ± 1.4 years; body height: 174.1 ± 6.0 cm; body mass: 75.5 ± 10.7 kg) were assigned to a randomized, placebo-controlled, double-blind trial of parallel group. Participants received either EMIQ in whey protein (EW, *n* = 19) or contrast whey protein (W, *n* = 20) 6 days per week over 4 months. Body composition was assessed using dual-energy X-ray absorptiometry. Markers of oxidative stress, derivatives of reactive oxygen metabolites (d-ROMs) and biological antioxidant potential (BAP), were assessed using a free radical analytical system. Data were analyzed using a univariate and repeated measures general model statistics.

**Results:**

After 4 months, changes in lower limb fat-free mass and muscle mass were significantly greater in the EW group than in the W group (mean change ±95% CI; W: 324.1 ± 284.3, EW: 950.3 ± 473.2, *p* = 0.031, W: 255.7 ± 288.6, EW: 930.9 ± 471.5, *p* = 0.021, respectively). Moreover, the EW group exhibited a significantly higher BAP/d-ROMs ratio, antioxidation index, than the W group after 4 months (mean change ± SD; W: 8.8 ± 1.1, EW: 10.3 ± 2.8; *p* = 0.028). No significant differences in body mass, lean body mass, fat mass, or lower limb fat mass were observed between the groups.

**Conclusion:**

Ingestion of EMIQ in supplementary protein powder for 4 months exerts antioxidant effects and increases muscle mass among American football players.

**Trial registration:**

University Hospital Medical Information Network Clinical Trial Registry, UMIN000036036. Retrospectively registered in 2019.

## Background

Rutin is a polyphenolic flavonoid compound found in many plants (e.g., citrus fruits, onions, and buckwheat) and exhibits strong antioxidant effects [1]. Rutin has vitamin-like and medicine-like effects, including preventing cardiovascular and metabolic diseases [2]. However, it is difficult for humans to absorb rutin [3]; therefore, it is converted to quercetin and its metabolites prior to absorption.

Enzymatically modified isoquercitrin (EMIQ), enzymatically modified rutin, and α-glycosyl isoquercitrin are glucosyl derivatives of quercetin. EMIQ is manufactured from rutin through enzymatic modification. The enzymatic treatment increases the water solubility of EMIQ; the bioavailability of EMIQ is approximately 17 times that of quercetin and 45 times that of rutin [4]. Compared to rutin, EMIQ is also more easily absorbed in humans [5]. Therefore, EMIQ is expected to provide greater health benefits than quercetin and rutin. EMIQ is approved as a safe food additive and an antioxidant [6, 7].

Mukai et al. reported that quercetin has a preventive effect on muscle atrophy [8]. They have reported that quercetin injection in rats suppressed the reduction of muscle mass by attenuating the induction of ubiquitin ligase. They also found that quercetin prevented muscle atrophy by targeting mitochondrial function [9]. Leelayuwat et al. reported that quercetin administration increased the cross-sectional area of skeletal muscles in swimming rats and promoted recovery from muscle inflammation after severe exercise [10]. Moreover, we found that EMIQ, a water-soluble quercetin, intensified muscle hypertrophy in mice [11]. Therefore, we hypothesized that supplementation with EMIQ will produce positive changes in human muscles as well.

Oxidative stress is closely related to exercise. Moderate exercise increases oxidative stress, prompting muscle hypertrophy [12]; whereas intense and prolonged exercise causes high levels of reactive oxygen species, prompting muscle weakness and fatigue [13, 14]. Derivatives of reactive oxygen metabolites (d-ROMs) and biological antioxidant potential (BAP) were used as oxidative stress markers in a clinical study [15]. d-ROMs indicate oxidative stress in blood samples; BAP indicates antioxidant ability, and the BAP/d-ROMs ratio represents the antioxidation index. A previous study reported that BAP/d-ROMs ratio was positively correlated with performance increase [16]; however the effects of antioxidants on muscle mass are not well understood.

Muscle protein constantly undergoes synthesis and breakdown, and skeletal muscle mass is regulated by many signals [17], including exercise, physical tension, nutrients, hormones, and cytokines [18]. Increased protein synthesis rather than the breakdown of muscle protein, causes increase muscle mass [19]. After exercising in the fasting state, the net muscle protein balance becomes negative, whereas when protein is ingested after the exercise, the net muscle protein balance becomes positive [20]. Therefore, many athletes use supplementary protein powder to gain skeletal muscle mass [21, 22].

In the present study, we examined the effects of the consumption of EMIQ on body composition and antioxidant status of American football players who undergo strong exercise training regularly. They typically consume supplementary protein powder, and EMIQ is supplied with protein powder. We hypothesized that EMIQ treatment will significantly increase lean mass and lower limb muscle mass. We further hypothesized that EMIQ treatment will significantly affect the antioxidant status.

## Methods

### Study design and participants

This randomized, parallel arm, placebo-controlled, double-blind study was conducted for 4 months in 2014. Primary outcomes were changes in lean mass and lower limb muscle mass, and secondary outcomes were changes in the antioxidant status. The study’s protocol was approved by the Ethics Committee of Tsukuba University (2014.7.30, no. 26–37), and the study was performed in accordance with the guidelines of the Declaration of Helsinki Declaration and the 2010 Consolidated Standards of Reporting Trials statement [23]. The trial was retrospectively registered in the University Hospital Medical Information Network Clinical Trial Registry (Japan, registration no. UMIN000036036) in 2019. Forty male Japanese students (from the Tsukuba University) who played American football (BMI ≥18.5 and < 30) were recruited to participate in the study. All participants provided written informed consent and were assigned to receive either EMIQ in whey protein (the EW group) or contrast whey protein (the W group), and the groups were stratified according to grade (junior vs. senior) and football position (back vs. lineman) using the “rand” function of Microsoft Excel (version 14.4.7). Time limits of whey protein powder with/without EMIQ are 18 months, and the protein was consumed within this limit. During the study period, the groups were identified as group A (the W group) and group B (the EW group) to ensure blinding.

### Exclusion criteria

The following participants were excluded:

1. Participants with food allergies.
2. Participants who consume other supplementary protein powders, drugs, or supplements during the study.
3. Participants who change their lifestyle, including dietary and exercise habits, during the study.
4. Participants who eat unbalanced diet (consuming much polyphenol-rich food, including citrus fruit, buckwheat, and fermented soybeans), or consume excessive alcohol.
5. Participants who refrained from practice for long periods (e.g., because of injury).

One of the recruited participants was excluded because he failed to provide a blood sample.

### Characteristics of participants and training program

**Fig. 1:**
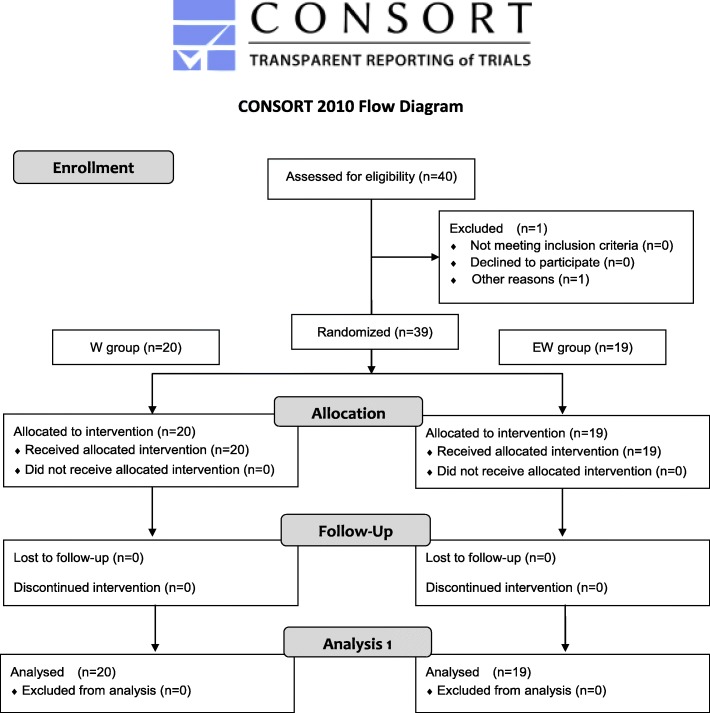
Study flow chart. Forty young male Japanese university students who played American football were recruited, although one participant was excluded because he did not provide blood sample. Thus, 39 participants were randomized to receive either whey protein (W) or EMIQ in whey protein (EW)

**Fig. 2:**
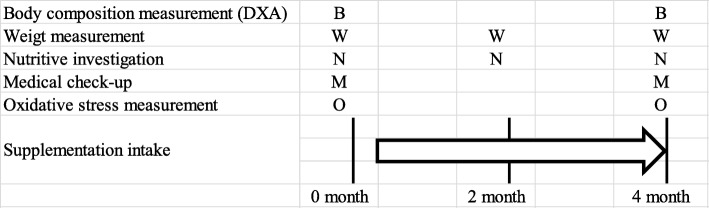
Study protocol. Participants consumed 20 g of their supplementary protein powder after exercise (6 days a week). Body composition was measured using dual-energy X-ray absorptiometry (DXA) at 0 and 4 months. Weight measurements and nutritional evaluations were performed at 0, 2, and 4 months. Medical check-ups were performed with blood sampling at 0 and 4 months. Oxidative stress was measured at 0 and 4 months. B: body composition measurement, W: weight measurement, N: nutritional evaluation, M: medical check-up, O: oxidative stress measurement

Most participants had previously consumed supplementary protein powder (typically whey protein, 3–4 times a week after resistance training). The study’s flow chart and assessment schedule are shown in Figs. 1 and 2, respectively.

The football team’s trainer provided input regarding the design of the training program. Resistance training to maintain or increase skeletal muscle mass and power was performed 3 times a week during the first month (from 0 month to 1 month). Participants also underwent skill training to increase their individual performance and teamwork. From 1 month to 2 month, the participants underwent moderately intense training (to develop physical strength and complete a training camp). From 2 month to 4 month, practice sessions/games were conducted 5–6 times a week (the competitive season).

### Supplementation protocols

Participants consumed 20 g per day of whey supplementary protein powder (the W group) or 20 g of whey supplementary protein powder with 42 mg of EMIQ (the EW group). As calculated, participants consumed 0.26 g/kg whey protein powder, and which indicates 0.18 g/kg protein. Consumption amount of protein was determined on the basis of previous participants’ habits and previous reports [24, 25]. All supplements were consumed 6 times a week (immediately after practice). Nutritional components of the supplements are shown in Tables 1 and 2. Moisture and mineral contents were 0.8 g and 0.5–0.96 g, respectively.

**Table 1:** Nutrition facts per 20 g supplementation

Per 20 g supplementation		
	W	EW
Energy	76 kcal	
Protein	13.3 g.	
Fat	0.14~0.60 g	
Carbohydrate	4.8 g	
EMIQ	–	42 mg

**Table 2:** Amino acid composition

(g/100 g supplement)	
Ala	3.44
Arg	1.49
Asp	7.47
Cys	3.2
Glu	12.4
Gly	1.17
His	1.24
Ile	4.26
Leu	7.12
Lys	6.24
Met	1.36
Phe	2.14
Pro	4.27
Ser	3.29
Thr	4.75
Typ	1.2
Tyr	1.91
Val	3.92

EMIQ in the supplements was analyzed using a previously described high-performance liquid chromatography method [26] that revealed that the whey protein powder contained 0.0 mg of EMIQ and whey protein powder with EMIQ contained 42 mg of EMIQ. All supplements were prepared by Morinaga & Co. Ltd. (Tokyo, Japan).

### Body composition measurements

Body weights were measured using a body composition meter (MC-190; TANITA, Japan) at baseline and 4 months. Body composition parameters (bone mineral content, fat mass, and muscle mass) were measured using dual-energy X-ray absorptiometry (DXA; QDR-4500A; Hologic, Japan) at baseline and 4 months. DXA measurements were conducted following overnight fast and 24-h absence of strenuous exercise. Participants wore typical athletic clothing and removed all metal jewelry. Participants were laid on their back on the DXA table with their arms at their sides and feet together. The same investigator conducted all analyses, and the second measurement was performed as a comparable mode. The investigator checked the setting of analysis region. Lower limb were separated from the trunk by a horizontal line just below the lower pelvic. Analysis provided data on lean body mass, fat mass, and bone mineral content for the total body and lower limb. Lower limb fat-free mass was calculated from lower limb lean mass plus lower limb bone mineral content. Lower limb lean mass was expressed as lower limb muscle mass because lower limbs consist mostly of skeletal muscle, bone, and fat [27].

### Nutritional evaluations

To assess food intake, participants completed food frequency questionnaires (FFQg version 3.5; Kenpaku-sha, Tokyo, Japan) at baseline, 2 months, and 4 months (on the same day as their body weight measurements). Supplementation with protein powder for this study was not included in these nutritional evaluations.

### Blood sampling

Blood sampling was performed at baseline and 4 months. The venous blood samples were collected in vacutainers in the morning after overnight fasting and 24-h absence of strenuous exercise; 2 mL was drawn for general testing and 9 mL for liver and renal function testing. All blood samples were analyzed at the Tsukuba i-Laboratory. The blood test parameters were red blood cell (RBC), and white blood cell (WBC) counts, hemoglobin (Hb), hematocrit (Ht), mean corpuscular volume (MCV), mean corpuscular hemoglobin (MCH), mean corpuscular hemoglobin concentration (MCHC), total bilirubin (T-BIL), creatinine (Crea), uric acid (UA), urea nitrogen (UN), aspartate transaminase (AST), alanine transaminase (ALT), lactate dehydrogenase (LDH), platelet (PLT), and γ-glutamyl transpeptidase (γ-GTP). RBC and WBC were measured using the electric resistance method. Hb was measured using the sodium lauryl sulfate-Hb method. Ht was measured using the RBC pulse peak method. MCV was calculated as follows: Ht(%)/RBC (10^6^/ mm^3^) × 10. MCHC was calculated as follows: Hb(g/dL)/Ht (%) × 100. T-BIL, Crea, and UA were measured using the enzymatic method. UN was measured the urease-UV method. AST, ALT, LDH, PLT, and γ-GTP were measured the Japan Society of Clinical Chemistry standardization method.

### Oxidative stress analysis

Blood samples were collected from participants’ fingertips in the morning after overnight fasting and 24-h absence of strenuous exercise at 0 and 4 months. The d-ROMs were measured using a free radical system (FRAS4; Health & Diagnostics Ltd., Italy) and measurement kits (DIACRON, Italy). The d-ROMs results were expressed in arbitrary units, with one unit corresponding to 0.08 mg/dL of hydrogen peroxide. BAP was also measured using the FRAS4 system and DIACRON measurement kits. The BAP results were expressed in mmol/L of reduced ferric ions.

### Statistical analysis

Data were expressed as mean ± standard deviation, and changed data were expressed as mean change ±95% CI. Data were analyzed using a general linear model (GLM) with repeated measures two-way analysis of variances (ANOVA), with two levels by time (pre- and post-test or pre-, 2 months, and post-test) and groups (W and EW) as the Levene’s test revealed homoscedasticity and the Kolmogorov-Smirnov test revealed normality. In some cases, simple main effect test was performed following repeated measures two-way ANOVA. Changed data were analyzed using a GLM with one-way ANOVA as the Levene’s test revealed homoscedasticity and the Kolmogorov-Smirnov test revealed normality. In addition, effect size (*ES*) was calculated with Cohen’s d as a standardized measurement based on SD differences. Values closer to 1 is indicated substantive significance. Statistical analyses were performed using the SPSS software (version 22.0; SPSS Inc., Chicago, IL, USA), and differences were considered statistically significant at *p* < 0.05.

## Results

### Nutrient intake

The FFQg questionnaire was used to evaluate nutrient intake; supplementary protein intake was not included in this questionnaire. There were no significant differences in nutrient intake differences during the study periods. There were no significant differences in nutrient intake during between the W and EW groups at 4 months, suggesting that dietary habits had no effect on the results (Table 2).

### Blood test results

Results of blood tests at baseline and 4 months are shown in Table 3. One of the participants in the W group and three of those in the EW group could not provide blood samples at baseline because of not meeting the schedules. Only AST values were higher in the W group than in the EW group during the experiment. There was no significant changes due to EMIQ supplementation. RBC, and WBC counts, Hb, Ht, MCV, Crea, ALT, PLT, and γ-GTP increased from baseline to 4 months, possibly because of the strenuous training. However, the participants exhibited no significant deviations from the reference ranges for blood parameters during the study period. No adverse events were associated with the investigational product during the study period.

**Table 3:** Nutrient intakes

		W group (*n* = 20)	EW group (*n* = 19)	interaction	*p* value
Energy	Baseline	38 ± 15	43 ± 16	Group	0.168
kcal/kg BW)	2 months	36 ± 14	41 ± 13	Time	0.065
	4 months	35 ± 10	38 ± 14	Group×Time	0.892
Protein	Baseline	1.1 ± 0.4	1.3 ± 0.5	Group	0.263
g/kg BW)	2 months	1.1 ± 0.4	1.2 ± 0.4	Time	0.156
	4 months	1.1 ± 0.4	1.2 ± 0.4	Group×Time	0.681
Fat	Baseline	1.1 ± 0.4	1.3 ± 0.5	Group	0.273
g/kg BW)	2 months	1.1 ± 0.4	1.2 ± 0.4	Time	0.075
	4 months	1.1 ± 0.4	1.2 ± 0.5	Group×Time	0.729
Carbohydrate	Baseline	5.7 ± 2.5	6.2 ± 2.5	Group	0.375
g/kg BW)	2 months	5.3 ± 2.2	6.0 ± 2.2	Time	0.101
	4 months	5.1 ± 1.5	5.5 ± 2.1	Group×Time	0.874

Data are mean ± SD; BW body weight

### Body composition results

**Fig. 3:**
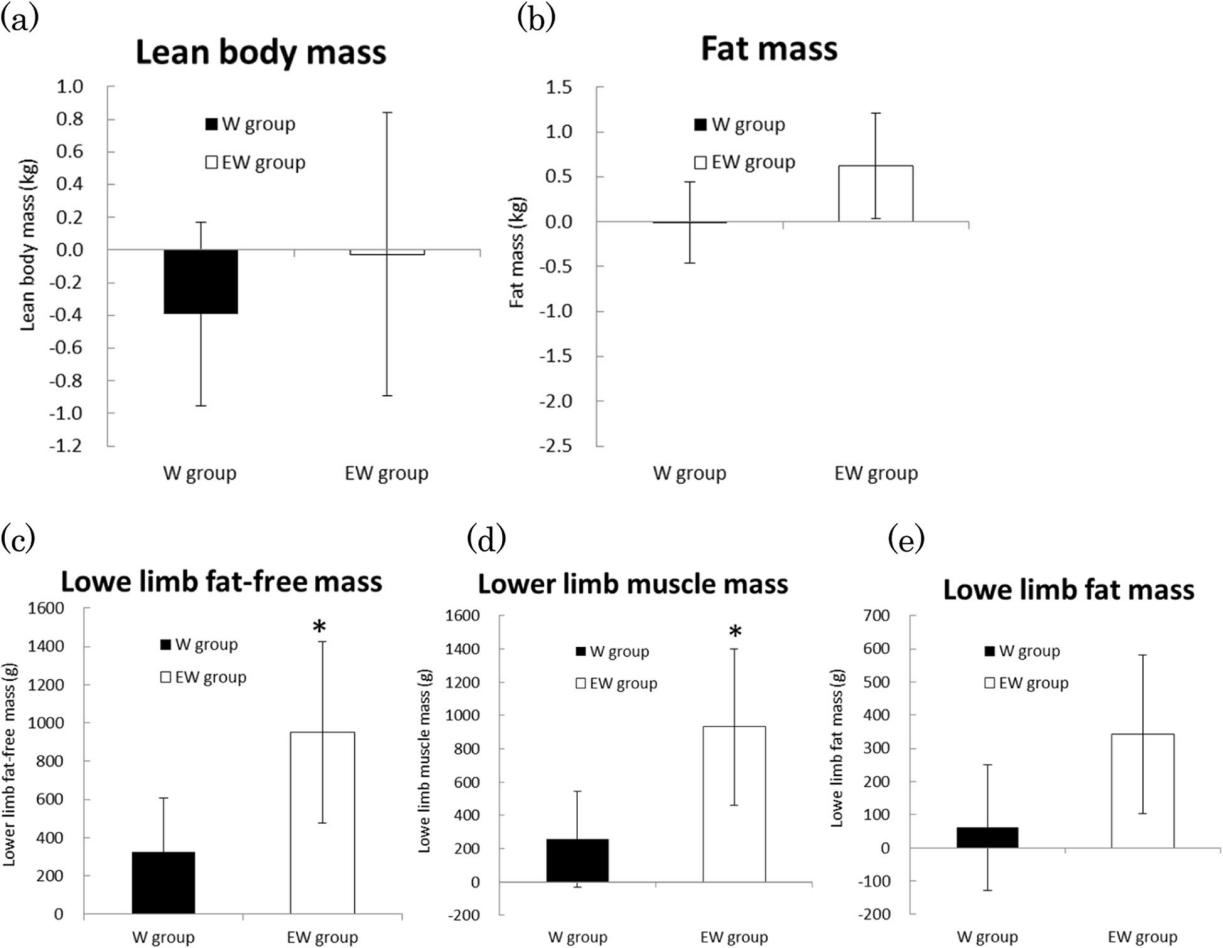
Lean body mass (**a**), fat mass (**b**), lower limb fat-free mass (**c**), lower limb muscle mass (**d**), and lower limb fat mass (**e**). Participants consumed 20 g of their supplementary protein powder after exercise (6 days a week). Changes of body composition from 0 to 4 months were expressed as mean change ±95% CI, and changes completely above or below the baseline are significant changes. *p < 0.05, significant difference between W group and EW group

Basic information of the two groups is shown in Table 4, and body composition data at baseline and 4 months are shown in Table 5. There were no differences in the basic information between the two groups. Lower limb fat-free mass, lower limb muscle mass, and lower limb fat mass increased from baseline to 4 months, possibly because of the strenuous training and protein consumption. Increase in lower limb fat-free mass and lower limb muscle mass was markedly observed in the EW group (Group × Time interaction). Changes in the body composition from baseline to 4 months are shown in Fig. 3. Increases in lower limb fat-free and muscle masses were significantly greater in the EW group than in the W group (*p* = 0.030, and 0.020, respectively), with a large effect size (*SE* = 0.740, and 0.800, respectively). There were no significant differences in changes in lean body mass, fat mass and lower limb fat mass between the groups.

**Table 4:** Blood test results

		W group (*n* = 20)		EW group (*n* = 19)		Interaction *p* value		
		Baseline	4 months	Baseline	4 months	Group	Time	Group×Tim
RBC	(× 10^5^/μL)	5.0 ± 0.3	5.3 ± 0.3	5.0 ± 0.3	5.3 ± 0.4	0.311	**0.003**	0.260
WBC	(× 1000/μL)	6.9 ± 1.2	7.7 ± 1.8	6.2 ± 1.1	7.1 ± 1.3	0.218	**0.001**	0.641
Hb	(g/dL)	15.4 ± 0.8	15.6 ± 0.6	15.6 ± 0.8	15.9 ± 0.9	0.477	**0.016**	0.244
Ht	(%)	44.2 ± 2.1	46.2 ± 1.7	44.8 ± 2.2	46.7 ± 2.7	0.441	**0.003**	0.253
MCV	(fL)	87.5 ± 2.5	87.6 ± 2.2	88.9 ± 3.0	88.9 ± 2.5	0.433	**0.040**	0.275
MCH	(pg)	30.4 ± 1.2	29.6 ± 1.1	30.9 ± 0.9	30.2 ± 0.9	0.472	0.119	0.266
MCHC	(%)	34.8 ± 0.6	33.8 ± 0.7	30.9 ± 10.7	34.0 ± 0.7	0.308	0.124	0.269
T-BIL	(mg/dL)	0.9 ± 0.5	0.7 ± 0.4	0.9 ± 0.4	0.7 ± 0.4	0.757	0.362	0.398
Crea	(mg/dL)	0.9 ± 0.1	0.9 ± 0.1	0.9 ± 0.1	0.9 ± 0.1	0.807	**0.033**	0.470
UA	(mg/dL)	6.1 ± 0.9	6.2 ± 1.2	5.9 ± 1.0	6.0 ± 1.2	0.212	0.069	0.370
UN	(mg/dL)	154 ± 4.4	15.2 ± 3.9	16.1 ± 2.9	16.1 ± 2.3	0.986	0.165	0.366
AST	(IU/L)	25.6 ± 5.8	24.1 ± 5.0	22.2 ± 4.4	22.5 ± 5.3	**0.045**	0.236	0.167
ALT	(IU/L)	20.4 ± 9.7	21.8 ± 9.6	19.6 ± 8.8	26.3 ± 13.5	0.783	**0.003**	0.063
LDH	(IU/L)	224.5 ± 30.6	192.8 ± 28.0	217.2 ± 45.7	187.1 ± 30.4	0.187	0.523	0.338
PLT	(×1000/μL)	245.8 ± 48.6	250.4 ± 50.4	246.2 ± 29.7	251.9 ± 35.7	0.508	**0.010**	0.225
γ-GTP	(IU/L)	20.4 ± 8.1	20.5 ± 5.3	19.8 ± 6.3	23.0 ± 6.8	0.966	**0.019**	0.092

Data are mean ± SD; *p* < 0.05 is considered significant (indicated in bold)

*RBC* red blood cell, *WBC* white blood cell, *Hb* hemoglobin, *Ht* hematocrit, *MCV* mean corpuscular volume, *MCH* mean corpuscular hemoglobin, *MCHC* mean corpuscular hemoglobin concentration, *T-BIL* total bilirubin, *Crea* creatinine, *UA* uric acid, *UN* urea nitrogen, *AST* aspartate transaminase, *ALT* alanine transaminase, *LDH* lactate dehydrogenase, *PLT* platelets, *γ-GTP* γ-glutamyl transpeptidase

**Table 5:** Basic information

	W group (*n* = 20)	EW group (*n* = 19)	
	Mean ± SD	Mean ± SD	*p* value
Age (year)	19.7 ± 1.3	20.0 ± 1.5	0.440
Body height (cm)	173.7 ± 6.2	174.6 ± 5.8	0.631

Data are mean ± SD; *BW* body weighta

### Antioxidant measurements

Antioxidant measurements are shown in Table 6. The EW and W groups did not exhibit any significant differences in BAP and d-ROMs at baseline. From baseline to 4 months, d-ROMs increased, indicating that the strenuous training increased plasma d-ROMs. Group interaction of BAP/d-ROMs showed 0.075; therefore, we analyzed simple main effect of groups. BAP/d-ROMs ratio at 4 months was significantly higher in the EW group than in the W group (*p* = 0.028, and *ES* = 0.750) Table 7.

**Table 6:** Body composition data from baseline to 4 month

	Group	Baseline	4 months	*p* value	
		Mean ± SD	Mean ± SD	Time	Group×Time
Body weight (kg)	W group	76.1 ± 10.2	75.6 ± 11.1	*0.743*	0.150
	EW group	74.9 ± 11.5	75.6 ± 12.1		
BMI (kg/m2)	W group	25.2 ± 2.6	25.0 ± 2.8	0.787	0.126
	EW group	24.5 ± 2.8	24.7 ± 3.0		
Lean body mass (kg)	W group	62.8 ± 7.5	62.4 ± 7.8	0.427	0.645
	EW group	61.7 ± 7.1	61.6 ± 7.3		
FAT mass (kg)	W group	11.2 ± 4.0	11.2 ± 4.3	0.108	0.101
	EW group	11.5 ± 4.9	12.1 ± 5.1		
Lower limb fat-free mass (g)	W group	22,710.0 ± 2854.4	23,034.0 ± 2993.4	**0.000**	**0.030**
	EW group	21,751.4 ± 2915.6	22,701.6 ± 3107.7		
Lower limb muscle mass (g)	W group	21,608.5 ± 2708.3	21,864.2 ± 2862.7	**0.000**	**0.020**
	EW group	20,635.9 ± 2811.6	21,566.7 ± 3005.3		
Lower limb fat mass (g)	W group	4368.8 ± 1751.9	4430.7 ± 1798.0	**0.013**	0.078
	EW group	4349.7 ± 1902.8	4692.0 ± 1998.7		

Data are mean ± SD; *p* < 0.05 is considered significant (indicated in bold)

**Table:** Table 7

	Group	Baseline	4 months	*p* value	
		Mean ± SD	Mean ± SD	Time	Group×Time
BAP (mmol/L)	W group	2068.6 ± 228.3	2027.1 ± 173.7	0.406	0.938
	EW group	2115.5 ± 340.1	2081.1 ± 208.0		
d-ROMs (U.CARR)	W group	249.1 ± 50.9	233.5 ± 34.7	**0.016**	0.884
	EW group	230.5 ± 57.2	213.1 ± 50.5		
BAP/d-ROMs	W group	8.7 ± 2.5	8.8 ± 1.1	0.230	0.328
	EW group	9.6 ± 2.4	10.3 ± 2.8*		

Data are mean ± SD; *p* < 0.05 is considered significant (indicated in bold)

* *p* < 0.05, significant difference between the W group and the EW group

U.CARR (Carratelli Units), where 1 U.CARR corresponds to 0.08 mg/dL hydrogen peroxide

## Discussion

The present study revealed that 4-month EMIQ supplementation in whey protein powder significantly increased lower limb lean mass in American football players. The EMIQ group also exhibited higher BAP/d-ROMs ratio than the control group after 4 months, suggesting that EMIQ supplementation improves the antioxidant status of players. To the best of our knowledge, this is the first report to show that antioxidants increase lower limb muscle mass, suppressing antioxidant stress.

During the experiments, body mass of players remained unchanged. Lower limb lean masses of both groups significantly increased between baseline and 4 months, possibly because of the training, a finding that is consistent with that of a previous report [28]. Lower limb muscle mass of participants in the EW group significantly increased from baseline to 4 months, whereas that of participants in the W group did not change significantly. Increases in lower limb lean mass and muscle mass were higher than in the EW group than in the W group. These results suggest that EMIQ supplementation may optimize the exercise effect and intensifies muscle hypertrophy in humans as they did in mice [11]. To clarify the detail mechanism of muscle hypertrophy due to EMIQ, further investigations will be needed.

One of the speculative mechanisms is suppression of oxidative stress. Antioxidant supplementation after exercise affects inflammatory markers, muscle fatigue and performance [29–31], although no report has suggested that antioxidant supplementation intensifies muscle hypertrophy. EMIQ supplementation may increase the endurance or work capacity of players, as revealed by studies that reported that quercetin improves muscle damage or endurance exercise capacity [32, 33]. However, we could not measure endurance or work capacity in this long-term study. Antioxidant status of a player is related to performance during fatiguing exercise [34, 35]; therefore, improvement of antioxidant status by EMIQ supplementation may be an advantage to players.

Another possible mechanism is the previously reported function of quercetin as an activator of mitochondria biogenesis and PGC-1α [36, 37]; these functions may affect muscle synthesis or breakdown [38, 39]. However, it is uncertain that the effect on atrophy and muscular dystrophy can be extrapolated to this small study [40]. In this study, EMIQ was consumed with supplementary protein powder, which may affect protein absorption and metabolism.

We observed no increases in the body weights in either group. As mentioned before, participants underwent training regularly and typically consumed supplementary protein powder. Therefore, it is possible that we did not observe significant increases in body weight and body lean mass during this period, in accordance with a previous study [41]. Meanwhile, some blood parameters mainly those related to inflammation and anemia, increased during the study, possibley because of the strenuous training. d-ROMs also increased during the study. These data indicate that during the study, all participants were under strenuous exercise and stress, with no differences between the groups.

In this study, we confirmed the safety of EMIQ. EMIQ is generally regarded as safe (GRAS) by the US Food and Drug Administration [7], and is approved as a food additive in Japan [6]. No adverse events were associated with EMIQ supplementation during the study period. Blood parameters did not alter with EMIQ supplementation, indicating that 42 mg of EMIQ supplementation is safe. Results of blood parameters revealed no health concerns in the study participants.

### Limitations

The present study evaluated only young male athletes because participants included were American football players of the university. Thus, there is a risk of age-related bias as age can be a factor affecting muscle hypertrophy. Furthermore, it remains unclear whether these results can be observed in individuals who exercise less frequently because exercise habits are also a factor. Further studies on other participants based on these limitations may clarify the effectiveness of EMIQ.

## Conclusions

Our findings suggest that EMIQ exerts antioxidant effects, improving lower limb muscle mass in American football players.

## Data Availability

Data are all contained within the article.

## Abbreviations

ALT: Alanine transaminase
AST: Aspartate transaminase
BAP: Biological antioxidant potential
BW: Body weight
Crea: Creatinine
d-ROMs: Derivatives of reactive oxygen metabolites
EMIQ: Enzymatically modified isoquercitrin
ES: Effect size
EW: Whey protein powder containing EMIQ
γ-GTP: γ-glutamyl transpeptidase
Ht: Hematocrit
Hb: Hemoglobin
LDH: Lactate dehydrogenase
MCH: Mean corpuscular hemoglobin
MCHC: Mean corpuscular hemoglobin concentration
MCV: Mean corpuscular volume
RBC: Red blood cell
T-BIL: Total bilirubin
UA: Uric acid
UN: Urea nitrogen
W: Whey protein powder
WBC: White blood cell
