# Correction to: Effects of enzymatically modified isoquercitrin in supplementary protein powder on athlete body composition: a randomized, placebo-controlled, doubleblind trial

**DOI:** 10.1186/s12970-019-0315-6

**Published:** 2019-10-21

**Authors:** Naomi Omi, Hideki Shiba, Eisaku Nishimura, Sakuka Tsukamoto, Hiroko Maruki-Uchida, Masaya Oda, Minoru Morita

**Affiliations:** 1 Faculty of Health and Sport Sciences, University of Tsukuba, Tsukuba, Japan; 2 Physical Education Graduate School, University of Tsukuba, Tsukuba, Japan; 3 Health Science Research Center, Morinaga & Co., Ltd, Tokyo, Japan

**Correction to: J Int Soc Sports Nutr**

**https://doi.org/10.1186/s12970-019-0303-x**

The original article [1] contained a typesetting error in Table 3 which was mistakenly introduced by the production team handling this article; this error has now been corrected.
